# Intelligent Distributed Optical Fiber Pressure Sensing for Dental Bite-Force Analysis

**DOI:** 10.3390/bios16090528

**Published:** 2026-09-21

**Authors:** Zhanerke Katrenova, Dauren Kussaiyn, Shakhrizat Alisherov, Wilfried Blanc, Alexandr Dostovalov, Amin Zollanvari, Carlo Molardi

**Affiliations:** 1 Department of Science and Innovation, Astana IT University, Astana 010000, Kazakhstan; 2 Department of Electrical and Computer Engineering, School of Engineering and Digital Sciences, Nazarbayev University, Astana 010000, Kazakhstan; 3 Institute de Physique de Nice, CNRS UMR7010, Université Côte d’Azur, Avenue Joseph Vallot, 06108 Nice, France; 4 Institute of Automation and Electrometry, Siberian Branch of the Russian Academy of Sciences, Novosibirsk 630090, Russia; 5 Physics Department, Novosibirsk State University, Novosibirsk 630090, Russia; 6 Department of Electrical and Computer Engineering, Utah Valley University, Orem, UT 84058, USA

**Keywords:** optical sensor, distributed sensing, SLMux, intelligent intraoral device, mouthguard, machine learning, dentistry

## Abstract

Measuring bite force is essential for assessing the masticatory system and diagnosing oral disease. Existing measurement devices have low spatial resolution and susceptibility to electromagnetic interference. This paper presents a machine learning (ML)-assisted distributed fiber optic sensing system based on Scattering Level Multiplexing (SLMux) for high-resolution bite force analysis. Enhanced backscattered data were acquired through optical backscattered reflectometry from 88 sensing points along the dental arch. Measured data were reconstructed into a two-dimensional map of bite force and analyzed through an ML pipeline. Sector classification across 4 regions and weight prediction were processed by an end-to-end fine-tuned ResNet-18 Convolutional Neural Network (CNN) and classical ML approaches. ResNet-18 is compared with Logistic Regression, Support Vector Machine (SVM), Random Forest, XGBoost (Extreme Gradient Boosting), Extra Trees, and k-Nearest Neighbors (kNN) trained on handcrafted features. On sector classification, Logistic Regression achieved the best performance (98.71% accuracy). On the weight prediction task, formulated as a 12-class problem, the end-to-end ResNet-18 CNN substantially outperformed all classical models, reaching 51.28% accuracy and a mean absolute error of 78 g, versus 124 g for the best classical model. A regression-based ResNet-18 variant was also trained on the wavelength-shift and weight data, resulting in a mean absolute error of 62.6 g. The results indicate that integrating ML with distributed fiber-optic sensing has the potential to enhance dental diagnostics and treatment planning.

## 1. Introduction

According to the World Health Organization (WHO), oral diseases affect over 3.7 billion people, diminishing the quality of life and complicating everyday procedures such as chewing and speaking [1]. A preventive mechanism for avoiding severe oral complications is monitoring abnormalities of the masticatory system. One of the key parameters that provides insights into the efficient performance of the mastication process is bite force measurement. High or low bite force values are associated with different oral diseases, such as periodontal issues and temporomandibular disorders (TMD), that can directly affect masticatory capacity [2,3,4]. Bite force measurement also serves to tune implant numbers, diameters, and designs to prevent overloading the dental jaw [5]. Assessment tools for measuring bite force rely on gauge transducers, piezoresistive transducers, piezoelectric transducers, and pressure-sensitive film. These techniques present limitations in terms of electromagnetic interference susceptibility, bulkiness, and low spatial resolution [6,7,8,9,10].

The fiber optic sensing devices are highly suitable for dental applications due to their non-electrical design for safe in vivo performance, metal-free composition for MRI compatibility, and multiplexing capabilities [11,12,13]. The main fiber optic sensor systems for assessing bite force mostly involve Fiber Bragg Grating (FBG) technology [14]. FBG consists of periodic modulation of the refractive index along the fiber core and has potential in dentistry due to its simple installation process and precise detection capabilities. Umesh et al. developed the FBG sensor, capable of measuring dynamic bite forces in living organisms [15]. Coimbra et al. applied chirped FBGs to implants and splints to analyze dental mechanics by measuring force intensity [16]. In addition to fiber-optic grating structures, distributed fiber-optic sensors were employed to measure the pattern of bite force across the entire jaw [17,18]. A distributed sensing system can offer densely located sensing points, allowing the reconstruction of an accurate map of dental forces [19,20]. Distributed fiber optic sensing devices may detect extremely weak signals along every segment of the fiber strand [19]. Rayleigh backscattering signals can be detected using optical backscattered reflectometry (OBR).

Rayleigh-based distributed sensing offers several advantages over standard grating systems, making it especially well-suited to bite-force mapping. First, a spatial resolution of up to 10 μm is necessary to rebuild a densely precise map of physical characteristics. FBG sensors often feature gratings several millimeters in length. Second, Rayleigh-based OBR offers a high optical dynamic range of 80 dB, which is far higher than normal FBG interrogators (usually 25–30 dB). Third, the number of FBG sensors multiplexed along a single fiber is limited by the wavelength range of the interrogator [20]. Rayleigh-based distributed sensing with OBR interrogation gives us the combined advantages of higher spatial resolution, higher sensitivity, and a much denser sensing grid [21,22].

OBRs are single-fiber systems that can only probe one fiber. In order to achieve distributed sensing along the surface, multiplexing is necessary. One of the novel technologies is Scattering Level Multiplexing (SLMux), which enables the simultaneous detection of several fibers by employing nanoparticle-doped fiber (NP-doped) [23,24,25].

The performance of fiber optic sensing systems can be significantly improved by applying ML techniques at various stages of the measurement pipeline [26,27,28]. ML algorithms learn complex, non-linear mappings from sensor signals to physical parameters without requiring explicit physical models. As a result, they are particularly well-suited for distributed sensing systems where the relationship between spatially resolved spectral data and measurands is inherently high-dimensional. In [29], Pal et al. demonstrated that Gaussian Process Regression applied to the FBG load cells might lead to weight measurement errors as low as 2.7 g, a 45% decrease in Root Mean Square Error (RMSE) in contrast to typical linear calibration. Shabalov et al. proposed a cascade ML architecture combining CatBoost classification with k-nearest neighbors regression for simultaneous force estimation, with a Mean Absolute Error (MAE) of 0.34 N (Newton) and impact localization (MAE = 2.1 mm) from FBG arrays, substantially outperforming rule-based baselines [30]. In dense multiplexed fiber networks, Manie et al. employed a Gated Recurrent Unit deep learning model to disentangle overlapping FBG spectra, providing higher density of sensing without accuracy loss [31]. Chen et al. applied a 1D CNN to fiber loop ring-down sensors, achieving about 98% accuracy of strain detection under harsh conditions [32]. These studies collectively demonstrate that ML can extract substantially more information from fiber-optic sensor data than traditional analytical approaches.

Convolutional Neural Networks (CNNs), in particular the pre-trained CNN architectures like ResNet and EfficientNet, have become well-established for most applications that require image-based classification or regression [33,34]. The utilization of transfer learning, which involves the initialization of model parameters using the trained weights from large-scale datasets such as ImageNet. Subsequently, exploiting the fine-tuning of the model to be specific to the dataset has been demonstrated to be highly effective, particularly in the absence of labeled training data. Therefore, it would make sense to transform the distributed fiber optic sensor data into 2D heatmaps to analyze them using pre-trained image classification networks.

Although there have been many studies where ML can enhance fiber optic sensing, this work is the first where ML is used with distributed fiber optic sensors for bite force measurement. The SLMux-based mouthguard system developed in the previous work demonstrated the sensor hardware using rule-based interpretation but did not explore ML-driven analysis [15,17]. Existing ML-enhanced fiber sensing studies employ fundamentally different sensor architectures (discrete FBG arrays rather than distributed Rayleigh scattering) and operate in domains other than dentistry. This represents a significant gap, as the high spatial density of distributed sensing generates rich datasets that are well-suited to ML-based pattern recognition but cannot be effectively analyzed using conventional peak-tracking or linear calibration methods.

This paper addresses this gap by developing and evaluating ML models for both force localization and force quantification using the SLMux-based mouthguard sensor. Raw OBR Rayleigh backscatter data from 88 measurement points across the dental arch are transformed into 2D heatmap images, bridging distributed fiber optic sensing with computer vision. Two complementary tasks are formulated: sector classification, which identifies the anatomical region (premolar or molar, left or right) where force was applied at 4-class granularity; and weight prediction, which estimates the applied force magnitude in grams as a 12-class classification problem and as a regression task that predicts the force directly in grams rather than in discrete units. For each task, an end-to-end fine-tuned ResNet-18 CNN is compared against classical ML—Logistic Regression, Random Forest, XGBoost, SVM, Extra Trees, and kNN [35,36,37]. This comparison provides a systematic evaluation of the contribution of end-to-end fine-tuning versus traditional classifiers for this novel sensor design based on the SLMux sensing system.

## 2. Methodology

### 2.1. Sensor System and Experimental Setup

**Figure 1:**
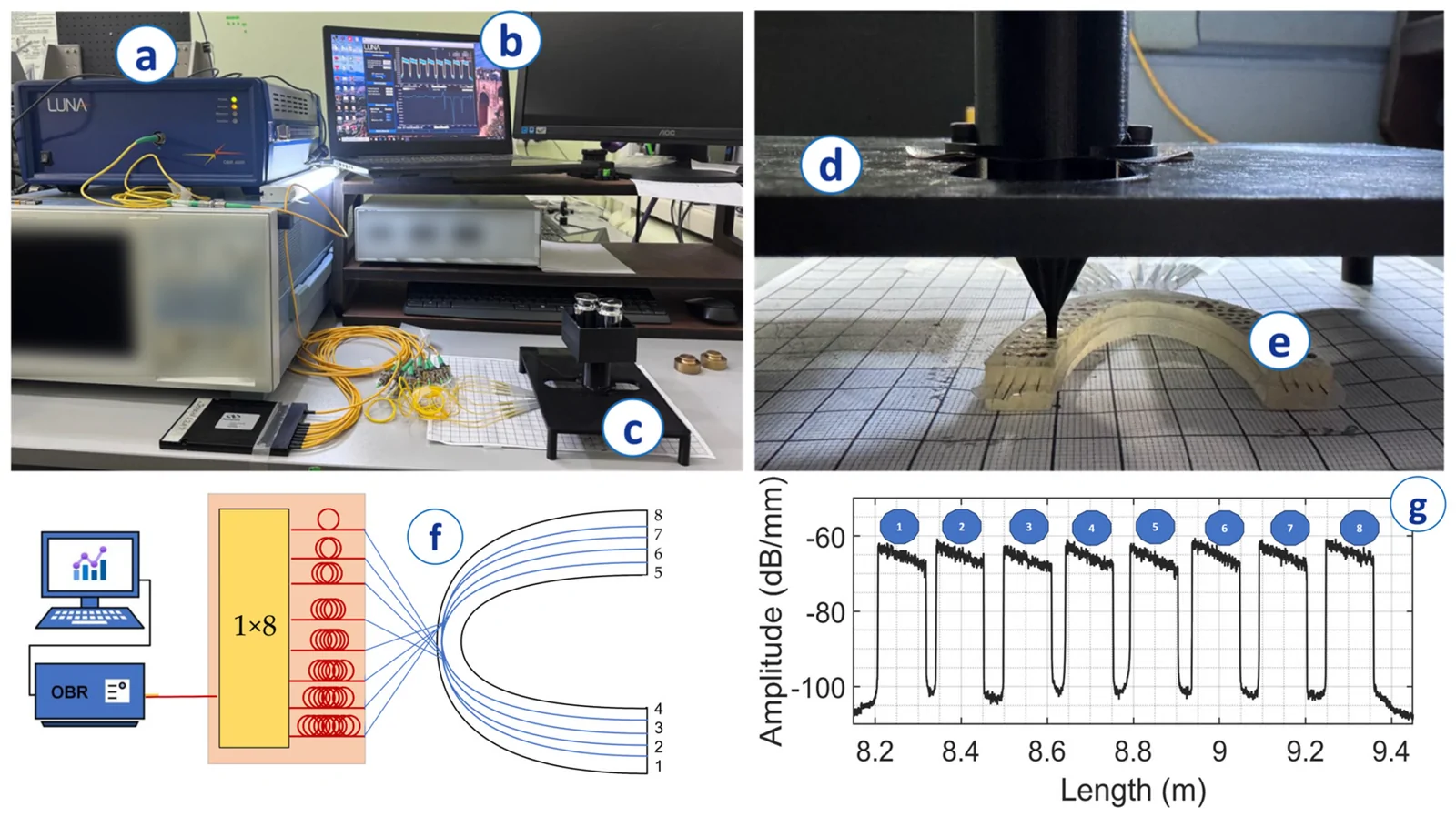
The experimental setup to collect data on 88 sensing points: (**a**) LUNA OBR interrogator; (**b**) computer software (OBR v3.13.0) for strain detection; (**c**) Rack structure with pressing tool; (**d**) Positioning pressing tool above silicone dental mouthguard; (**e**) Silicone dental mouthguard embedded with fiber optic sensing; (**f**) Connecting dental mouthguard to the LUNA OBR; (**g**) The scattering power of the SLMux configuration.

The experimental setup for data collection in the ML pipeline is shown in Figure 1. The fiber-optic sensing system developed by Katrenova et al. has been utilized to conduct bite-force mapping experiments [17]. To collect the data along the surface of the dental bite, a LUNA OBR 4600 (Luna Inc., Blacksburg, VA, USA) with strain-detection software (OBR v3.13.0) has been employed, as illustrated in Figure 1a,b. For applying point pressure, a 3D-printed rack structure and a pressing tool with a 2 mm tip diameter have been utilized, as shown in Figure 1c,d. The sensing system was created by embedding fiber lines into a custom dental mouthguard with an approximate height of 5 cm and a width of 6 cm, made with the silicone material Sorta Clear 18. The fiber-embedded dental mouthguard is shown in Figure 1e.

To achieve distributed sensing, the SLMux configuration was exploited. To cover the surface of the dental mouthguard, 8 parallel fiber lines were arranged across the sensing area, four on each side of the dental arch, each with a length of 6 cm. The schematic of the fiber organization inside the silicone mouthguard is illustrated in Figure 1f, where the red lines represent simple SMF-28, and the blue lines represent nanoparticle-doped fibers. Both fiber types have the same core (~8 µm) and cladding (125 µm) diameters, which allows for reliable splicing. All eight NP-doped fibers were spliced with different lengths of SMF-28, multiplexed through an 8-channel multiplexer, and connected to the interrogator. The configuration of these interconnected fibers provides the foundation of the SLMux architecture, which differentiates each sensing element by its backscattered signal and allows simultaneous scanning of all eight sensing fibers [23]. In this architecture, the standard SMF served as a separator, while the NP-doped fiber functioned as the sensing element, and the length of the SMF was set to differentiate each peak of the NP-doped fiber. The NP-doped fiber was drawn at 2050 °C from a preform prepared with the Modified Chemical Vapor Deposition process, using a high concentration of LaCl_3_ (0.7 mol/L) in the doping solution. During the drawing procedure, nanoparticles were formed, with a size of approximately less than 50 nm. Owing to the strong backscattered signal of the NP-doped fibers, distinct peaks are observed for each line, as demonstrated in Figure 1g, where the peaks on the power-intensity graph have been labeled according to each fiber line.

The left and right sides of the dental arch each occupied approximately 3 cm of the lateral width. The active sensing region used for reliable measurements was limited to the inner area between 0.5 and 4 cm vertically. Measurements outside this region were avoided because of the fiber-embedding architecture, which could otherwise affect the accuracy of localized pressure sensing.

**Figure 2:**
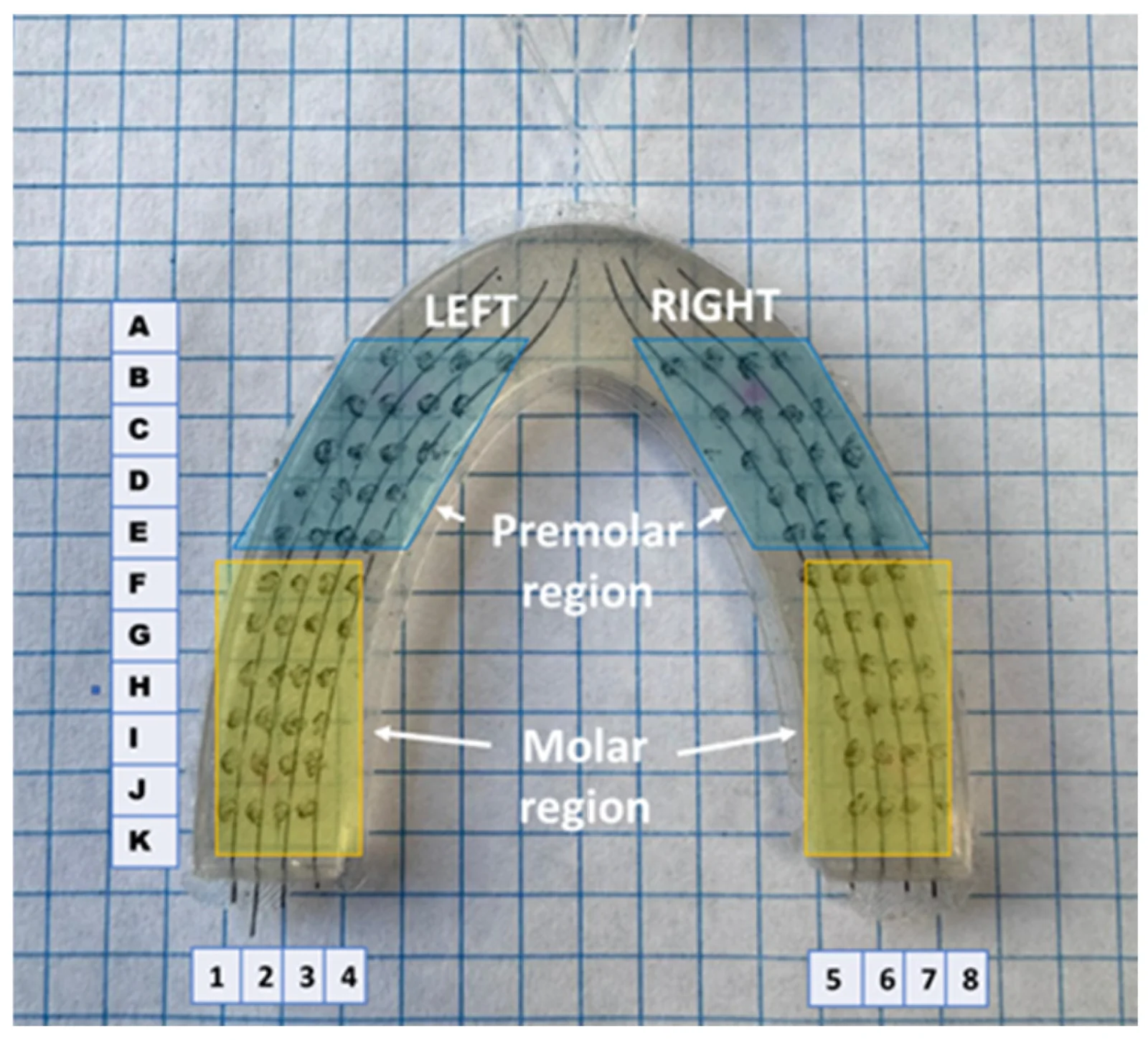
Anatomically divided regions of silicone dental model: left and right premolars (blue rectangles); left and right molars (yellow rectangles).

Calibration weights ranging from 100 g to 1200 g with a step of 100 g were used to apply pressure. To properly organize the application of pressure to sensing points, the silicone-based sensor was labeled into rows and columns. Figure 2 shows the labeling of the dental mouthguard by dividing the dental arch into 11 rows (labeled A through K, from the anterior premolar to the posterior molar region, where A–E corresponded to the premolar region and rows F–K corresponded to the molar region). The columns were labeled from 1 to 8 and corresponded to the positions of the optical fibers embedded in the dental mouthguard. The nominal spacing between adjacent columns was approximately 0.2 cm, while the spacing between adjacent rows was approximately 0.3 cm. Columns 1–4 correspond to the left side of the dental arch, while columns 5–8 correspond to the right side.

These coordinates were used as reference points for applying calibration weights and reconstructing the pressure distribution maps in MATLAB (R2025b). Since the weight was applied manually using a pressing tool, slight deviations from the marked positions were possible.

### 2.2. Data Acquisition and Preprocessing

Data collection was performed by systematically applying calibration weights at each of the 88 sensing points on the mouthguard surface. Figure 1a,c illustrate the experimental setup that consists of a LUNA OBR interrogator, software for strain detection, and a rack structure with a silicone-dental mouthguard, respectively. At each sensing point labeled on the dental mouthguard in Figure 2, 12 distinct weight values were applied sequentially: from 100 g to 1200 g with a step of 100 g. Before applying pressure, the reference state was recorded. For each weight-location combination, a calibration weight was placed on a cylindrical pressing tip (2 mm diameter) mounted on a custom rack structure and adjusted to a chosen sensing location, as shown in Figure 1d,e. Applied pressure as an external stimulus causes a shift in the wavelength value, which was measured as the difference between the reference and perturbed states of a dental mouthguard. The shift in wavelength is highly correlated with the applied value of mass or value of pressure [17]. The interrogator recorded the Rayleigh backscatter spectral data along all eight fiber branches as shown in Figure 1g. The results in tab-delimited text files containing wavelength shift versus fiber distance were saved. In total, 88 points × 12 weight levels = 1056 individual recordings in the OBR txt file were collected. After excluding sensing positions that lacked a complete set of usable recordings, 1019 heatmap images were retained for the machine-learning experiments.

From each OBR text file, 606 data lines, covering each parallel line of fiber embedded into silicone, were extracted. Further, the 2D array of fiber length and spectral shift values was processed through MATLAB (R2025b) software. The spectral shift values were converted to wavelength shifts. To isolate the signal from the eight active nanoparticle-doped sensing segments, a spectral filter was applied by zeroing wavelength shift values in predefined index ranges corresponding to non-sensing portions of the fiber (standard SMF-28). The remaining signal was segmented into eight fiber channels, each containing 31 data points representing the spatially resolved wavelength shift along approximately 6 cm of sensing fiber at 2 mm resolution.

After applying the pressure, the wavelength value is shifted, and the difference between the reference and perturbed states of the backscattered signal yields a wavelength shift that can be associated with the applied pressure. The file naming convention encodes both the weight and the sensing point coordinate (e.g., “600g_D3.txt”), enabling automated label extraction during the subsequent ML pipeline.

### 2.3. Machine Learning Framework

The ML pipeline addresses two complementary prediction tasks derived from the SLMux mouthguard data. The first task is sector classification, which identifies the anatomical region of the dental arch where force was applied. The second task is weight prediction, which estimates the magnitude of the applied force in grams. For each task, both an end-to-end fine-tuned CNN and traditional ML models utilizing handcrafted features were executed and carefully compared.

#### 2.3.1. Heatmap Generation

Raw OBR wavelength shift data from the eight fiber branches were transformed into two-dimensional heatmap images using a custom MATLAB (R2025b) pipeline. A spectral filter was applied to isolate the eight nanoparticle-doped sensing segments by zeroing non-sensing index ranges corresponding to standard fiber, splitter, and connector regions.

**Figure 3:**
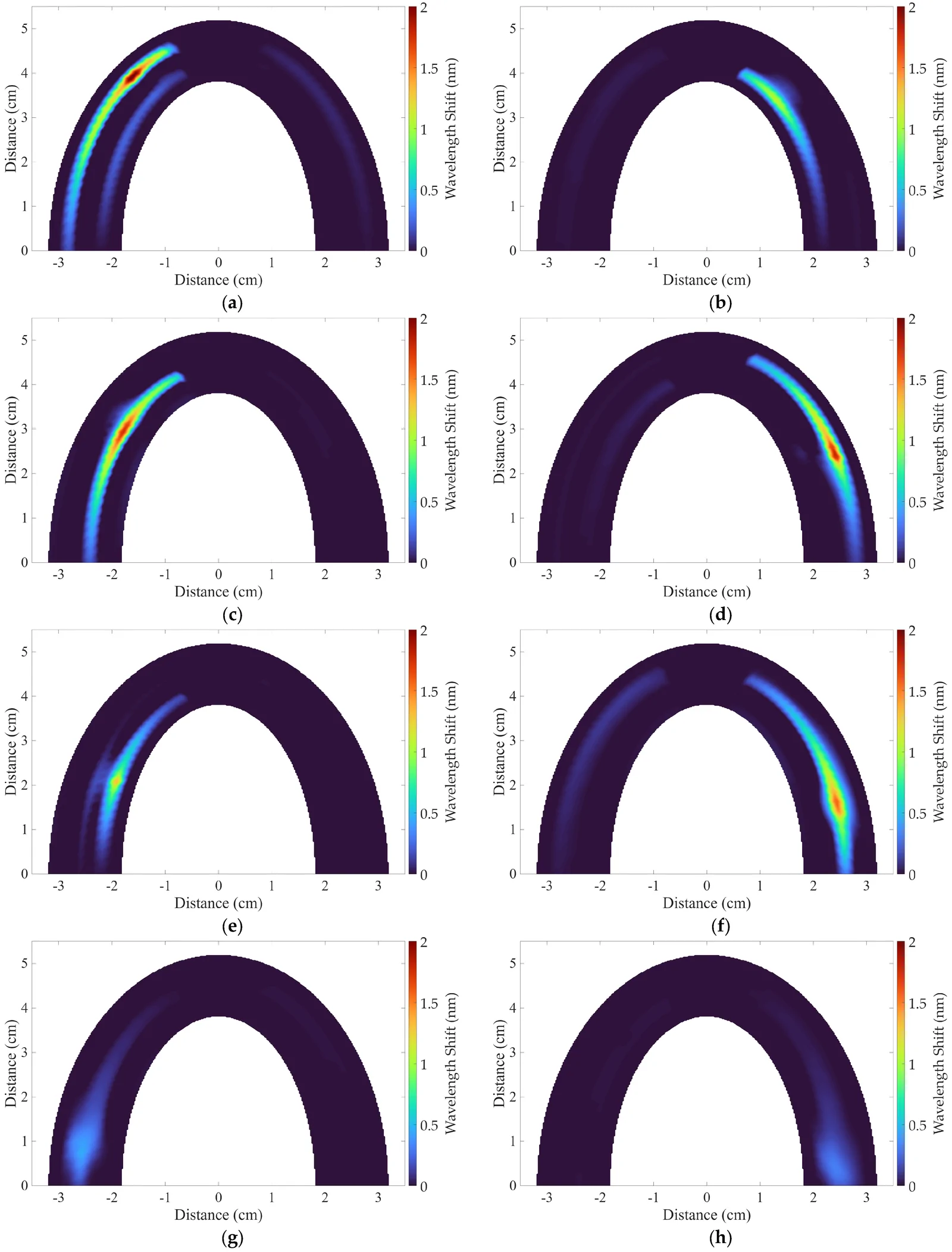
Reconstructed dental bite maps for localized loads applied at different sensing points: (**a**) A1—1200 g, (**b**) B5—600 g, (**c**) D3—900 g, (**d**) F8—1200 g, (**e**) G4—600 g, (**f**) H7—900 g, (**g**) J2—300 g, and (**h**) K6—300 g.

The filtered data from the eight fiber channels were mapped onto a semi-ellipsoidal coordinate system approximating the U-shape of the dental arch. Surface interpolation was performed using MATLAB’s Curve Fitting Toolbox with linear interpolation, and the resulting surface was rendered as a top-down 2D heatmap using the turbo colormap. The 2D maps of dental mouthguards are illustrated in Figure 3. The fixed color axis of [0, 2.0] nm was enforced across all 1019 heatmap images to ensure consistent absolute encoding of wavelength-shift magnitude and prevent auto-scaling artifacts. Each heatmap was exported as a 300 DPI PNG image with axis labels and grid lines suppressed.

#### 2.3.2. Task Formulation and Labeling

For sector classification, 1019 heatmap images were grouped by anatomical region as shown in Figure 3. In the 4-class formulation, sectors were defined as Left Premolar (rows A–E, columns 1–4), Left Molar (rows F–K, columns 1–4), Right Premolar (rows A–E, columns 5–8), and Right Molar (rows F–K, columns 5–8).

For weight prediction, a 12-class classification approach treating each calibration weight (100–1200 g) (trained with cross-entropy loss) was investigated.

#### 2.3.3. Dataset Preparation

The complete dataset of 1019 heatmap images was partitioned at the grid-cell (group) level into training (~70%), validation (~15%), and test (~15%) subsets with a fixed seed of 42. Each (row, column) sensing position was assigned in its entirety to a single subset, so that no position is shared across subsets; this leakage-aware grouping guarantees that every test grid cell is unseen during training and that a model cannot exploit position-specific artifacts learned at another weight. Labels were automatically extracted from filenames encoding both grid coordinate and applied weight. Both tasks used the identical group-level split, so that the held-out test cells coincide across all CNN and classical models and the results are directly comparable.

**Figure 4:**
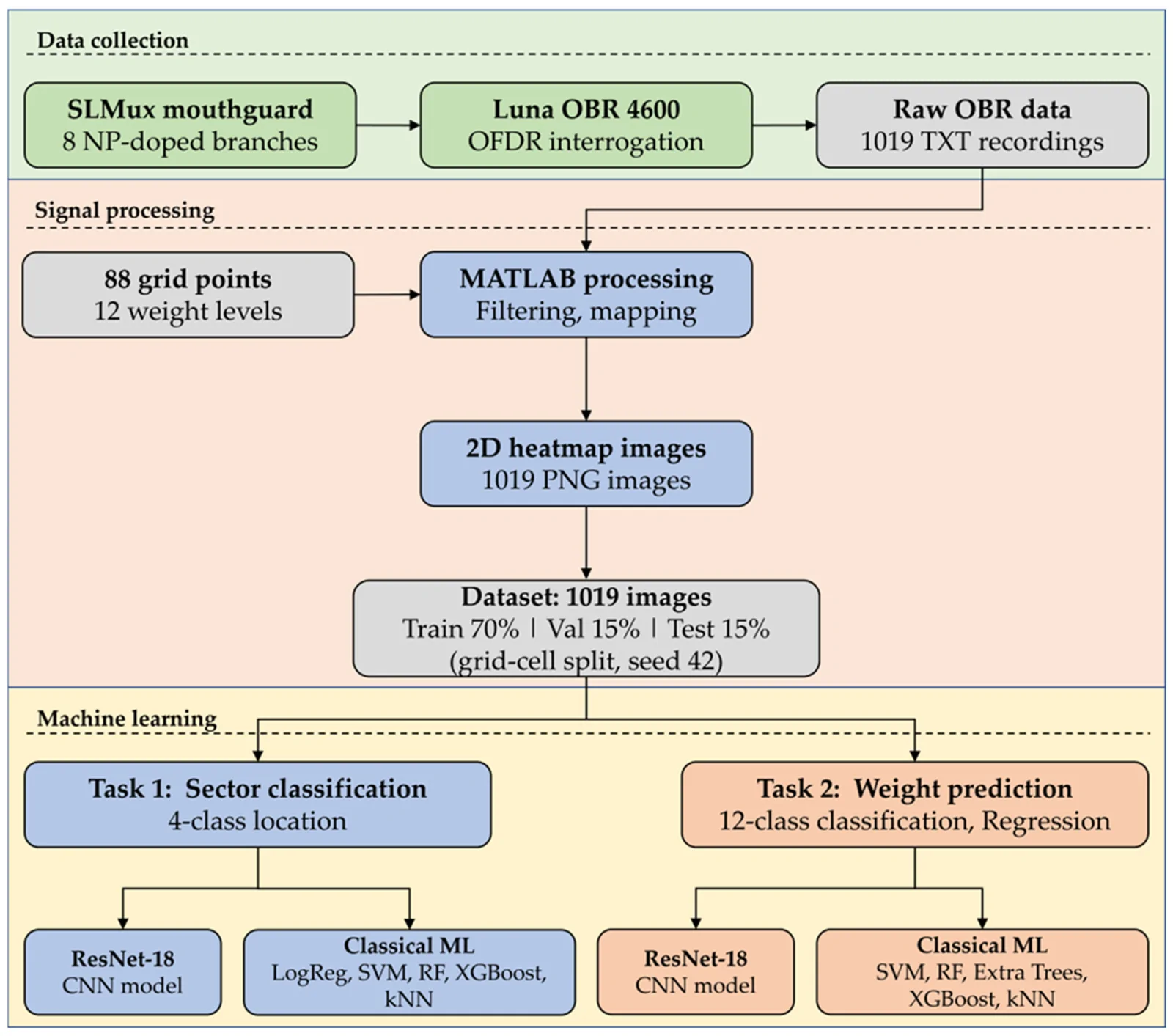
End-to-end methodology pipeline: from fiber optic data acquisition through signal processing and heatmap generation to machine learning-based sector classification and weight prediction.

For each task, two machine learning pipelines were implemented: deep learning and traditional machine learning approaches, including Logistic Regression, Random Forest, XGBoost, SVM, Extra Trees, and kNN [35,36,37]. Figure 4 illustrates the methodology pipeline starting from OBR data to ML tasks.

#### 2.3.4. Deep Learning Models

All heatmap images were resized to 224 × 224 pixels, converted to three-channel RGB tensors, and normalized using ImageNet statistics (mean = [0.485, 0.456, 0.406], std = [0.229, 0.224, 0.225]). Data augmentation during training consisted of random rotation (±8°, *p* = 0.7) and random affine transformations (3% translation, 95–105% scaling, *p* = 0.7). For weight prediction, random horizontal flipping (*p* = 0.5) and color jitter (brightness/contrast ±20%) were additionally applied.

The primary architecture employed a ResNet-18 backbone pretrained on ImageNet-1K. The original fully connected classification head was replaced with task-specific output layers, and the entire network—backbone and head—was then fine-tuned end-to-end on our heatmap data. No layers were frozen; the pretrained ImageNet weights served only as an initialization, allowing the convolutional filters to adapt to the statistics of fiber-optic heatmaps, which differ substantially from natural photographs. The output of ResNet-18’s final convolutional block is passed through global average pooling to obtain a 512-dimensional image feature vector that serves as the shared image representation.

Two prediction tasks were targeted—sector classification (where force is applied, 4 classes) and weight prediction (how much force is applied, 12 weight classes from 100 to 1200 g), as shown in Figure 4. Weight prediction was designed as a 12-class classification problem (100 to 1200 g in 100 g increments) and as a regression problem that directly outputs the applied force in grams. These tasks correspond to distinct deployment scenarios that can be run independently or in sequence. They are not redundant: an intraoral monitor would typically need both the location and the magnitude of each bite, obtained from the same heatmap frame.

All deep learning models were trained using the AdamW optimizer (learning rate = 3 × 10^−4^, weight decay = 1 × 10^−4^) with cosine annealing scheduling. Gradient clipping (max norm = 1.0) was applied, and training proceeded for up to 30 epochs with early stopping (patience = 8–10 epochs) based on validation macro-F1, evaluated on the held-out validation grid cells. The batch size was 16. The models were implemented in PyTorch 2.8.0 with torchvision 0.23.0.

#### 2.3.5. Classical Machine Learning Models

As classical ML approaches, five handcrafted-feature classifiers were applied to our data. The sector task used Logistic Regression, SVM with a radial basis function (RBF) kernel, Random Forest, XGBoost, and kNN; the weight task replaced Logistic Regression with Extra Trees, keeping SVM (RBF), Random Forest, XGBoost, and kNN [35,36,37]. No deep features were used: every heatmap was resized to a fixed 128 × 128 grid prior to feature extraction so that all samples shared a common spatial support. A handcrafted descriptor was computed directly from each 128 × 128 heatmap and flattened into a single feature vector, combining a histogram of oriented gradients (HOG), per-channel color histograms, a local binary pattern (LBP) texture histogram, and spatial grid statistics. The descriptor dimensionality was 2894 for the sector task and 8398 for the weight task, reflecting the different feature configurations used for each problem. Each classifier was wrapped in a pipeline (standardization, followed by PCA dimensionality reduction for the weight task, then the estimator), and hyperparameters were tuned by GroupKFold cross-validation on the train + validation (fit) grid cells, optimizing the macro-averaged F1-score; all preprocessing was refit inside each cross-validation fold so that no information leaked across folds. Classification quality is reported as test-set accuracy and macro-averaged F1-score, with the latter weighting every sector or weight class equally regardless of support. For the weight task, we additionally report the mean absolute error in grams (MAE), which credits predictions that fall on a neighboring load level and, therefore, conveys the ordinal severity of misclassification. Per-class precision, recall, and F1 are summarized for the best-performing sector model, and confusion matrices are provided for visual inspection.

Hyperparameters for each classifier were selected by GroupKFold (4-fold) cross-validated grid search on the train + validation (fit) grid cells, optimizing macro-F1; because grid cells define the cross-validation folds, no position is shared between a fold’s training and validation parts. The search grids covered: Random Forest and Extra Trees (n_estimators ∈ {300, 600}, max_depth ∈ {None, 20, 40}); SVM-RBF (C ∈ {1, 10, 100}, γ ∈ {scale, 0.01, 0.001}); kNN (k ∈ {3, 5, 7, 11}, weights ∈ {uniform, distance}); XGBoost (n_estimators ∈ {300, 600}, max_depth ∈ {4, 6}, learning_rate ∈ {0.05, 0.1}); and Logistic Regression (C ∈ {0.1, 1, 10}). For the weight task, the standardized features were additionally reduced by PCA (150 components) inside each fold. The best configuration per model was refit on the full fit (train + validation) set and evaluated once on the held-out test cells; the test set was not used at any stage of hyperparameter selection. The selected configurations were—sector task: Logistic Regression (C = 0.1), SVM (C = 1, γ = scale), Random Forest (600 trees, no depth limit), XGBoost (600 trees, learning_rate = 0.1, max_depth = 4), kNN (k = 11, uniform); weight task: SVM (C = 100, γ = scale), Random Forest (600 trees), Extra Trees (300 trees), XGBoost (300 trees, learning_rate = 0.1, max_depth = 6), kNN (k = 5, distance-weighted). All models were implemented using scikit-learn and XGBoost with a fixed random seed of 42. All models were implemented in scikit-learn 1.6.1 and XGBoost 2.1.4 under Python 3.9.6, and the handcrafted features (HOG, LBP) were extracted using scikit-image 0.24.0. A fixed random seed of 42 was applied uniformly to provide repeatability.

#### 2.3.6. Evaluation Metrics

For sector classification, overall accuracy, macro-averaged F1-score, per-class precision, recall, and F1-score, and confusion matrices were used. For weight classification, accuracy, macro-F1, MAE (obtained by mapping predicted class labels back to gram values), and the fraction of predictions within ±10% of the true weight were reported; MAE credits predictions that fall on a neighboring load level and therefore conveys the ordinal severity of misclassification. For the classical models, the GroupKFold cross-validation macro-F1 obtained during tuning is reported alongside the test metrics. All metrics were computed exclusively on the held-out test set. Table 1 presents a summary of the ML experimental settings.

## 3. Results

This section presents the experimental results for the two primary ML tasks: sector classification (localization) and weight prediction (force quantification). For each task, the end-to-end fine-tuned CNN is compared against classical ML models, with all models evaluated on a held-out test set under a rigorous group-level data split.

### 3.1. Sector Classification

For the 4-sector classification task, the end-to-end fine-tuned ResNet-18 CNN and five classical ML models—Logistic Regression, SVM (RBF kernel), Random Forest, XGBoost, and kNN—were trained on handcrafted statistical and spatial features extracted from the heatmaps. To ensure a rigorous and unbiased evaluation, all models were assessed using a group-level data split in which the (row, column) grid cells used for training, validation, and testing do not overlap; this prevents spatially adjacent measurements of the same location from leaking across the train/test boundary. Table 2 summarizes the test performance of all six models on the held-out group-level test set of 155 samples.

After hyperparameter tuning, the two strongest handcrafted-feature classifiers—Logistic Regression (98.71% accuracy, macro-F1 = 0.9869) and SVM (98.71%, macro-F1 = 0.9861)—slightly exceeded the end-to-end ResNet-18 CNN (98.06% accuracy, macro-F1 = 0.9807) on the 155-sample group-level test set, and Random Forest matched the CNN (98.06%, macro-F1 = 0.9805); XGBoost (95.48%, macro-F1 = 0.9520) and kNN (90.97%, macro-F1 = 0.8990) trailed. The selected hyperparameters were: Logistic Regression (C = 0.1); SVM (C = 1, γ = scale); Random Forest (600 trees, no depth limit); XGBoost (600 trees, learning rate = 0.1, max_depth = 4); kNN (k = 11, uniform). For every classical model, the held-out test macro-F1 (0.90–0.99) exceeded the GroupKFold cross-validation estimate obtained during tuning (0.75–0.84), as expected when each CV fold is trained on fewer grid cells than the final fit, indicating that the strong test performance is not an artifact of a particular split.

**Figure 5:**
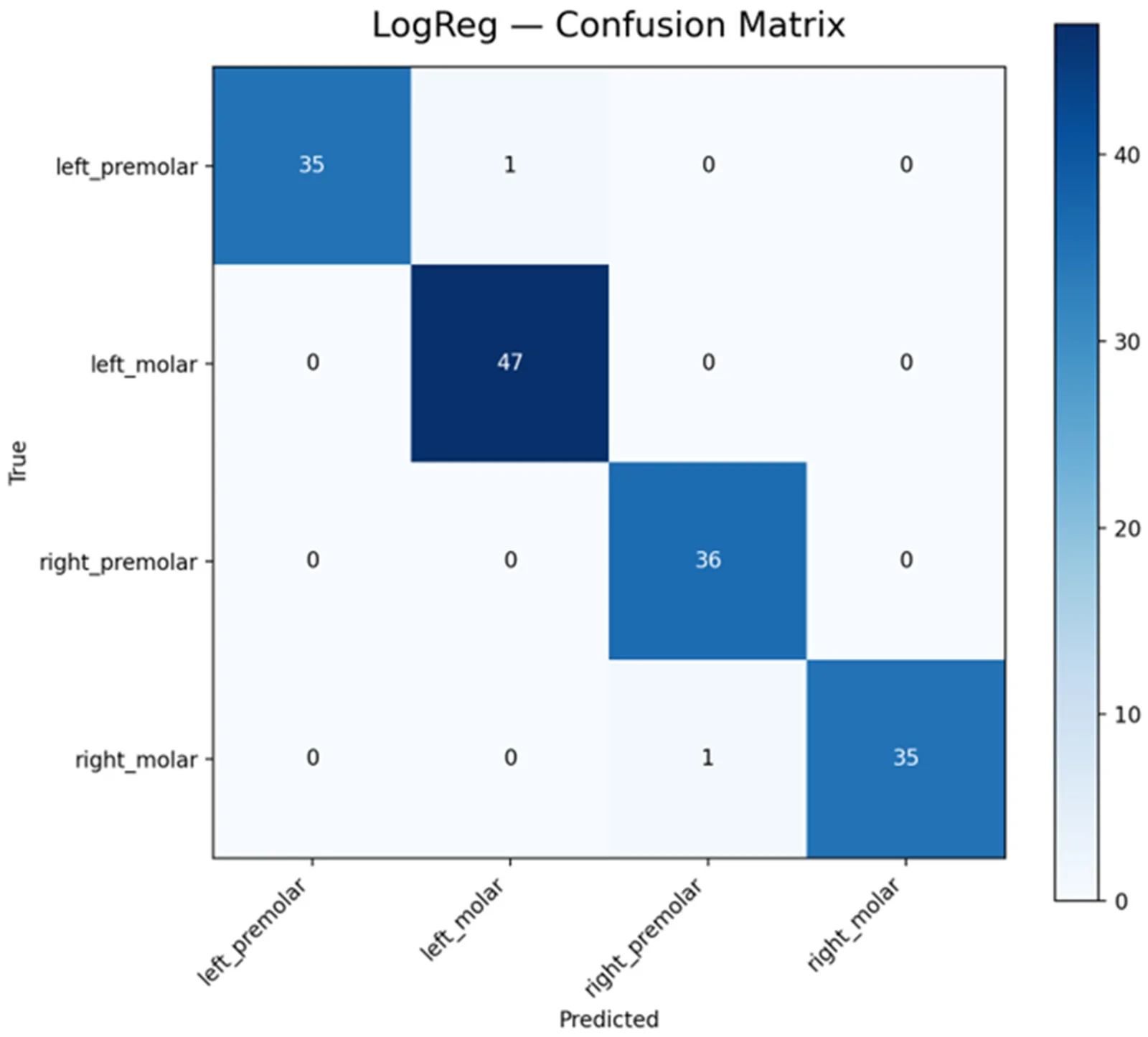
Confusion matrix for the 4-sector classification by Logistic Regression on handcrafted features.

As shown in Figure 5, Logistic Regression misclassified only two of 155 samples, both between adjacent regions of premolar and molar sectors; left–right confusion did not occur. The strongly diagonal matrix confirms that the handcrafted features reliably capture the spatial patterns needed for sector localization.

The end-to-end CNN (98.06% accuracy, macro-F1 = 0.9807) and the best handcrafted-feature classifiers—Logistic Regression and SVM (98.71%)—were effectively indistinguishable on the 4-class task, each making only two to three errors on the 155-sample group-level test set, and the residual misclassifications fell between spatially adjacent sectors. Because every test grid cell is unseen during training under the leakage-aware split, this parity reflects genuine generalization rather than memorization of position-specific artifacts.

The classical ML model Logistic Regression can achieve performance comparable to the much more complex ResNet-18. This outcome can be attributed to two main factors. First, the sector classification task mainly relies on the general position of the active region rather than small changes in intensity. Hand-crafted features such as HOG, color histograms, and spatial grids capture this information almost entirely. With these features, a simple linear decision boundary is sufficient, so a deep network provides no real benefit. The second reason is that ResNet-18 (about 11 M parameters) does not perform well on the group-level split because the data size is not enough to support such a large model, leading to high error variance. In contrast, Logistic Regression (with strong regularization, C = 0.1) can accept a little more bias in return for much lower variance, which is advantageous when there are only 713 training images. This bias–variance trade-off is the mechanism underlying the scissors effect [38]. The linear model is also far more practical for use with an intraoral device: its coefficients can be interpreted to indicate which spatial features are important, it uses considerably less memory and computing power than a CNN, and it can be reliably retrained each time. SVM (RBF) exhibited the same behavior and performed equally well. Tree-based models (Random Forest, XGBoost) and kNN did not perform as well, mainly because their methods are based on splitting along axes or relying on distance in high-dimensional space, which are less effective.

### 3.2. Weight Prediction

#### 3.2.1. Classification-Based Weight Prediction

**Figure 6:**
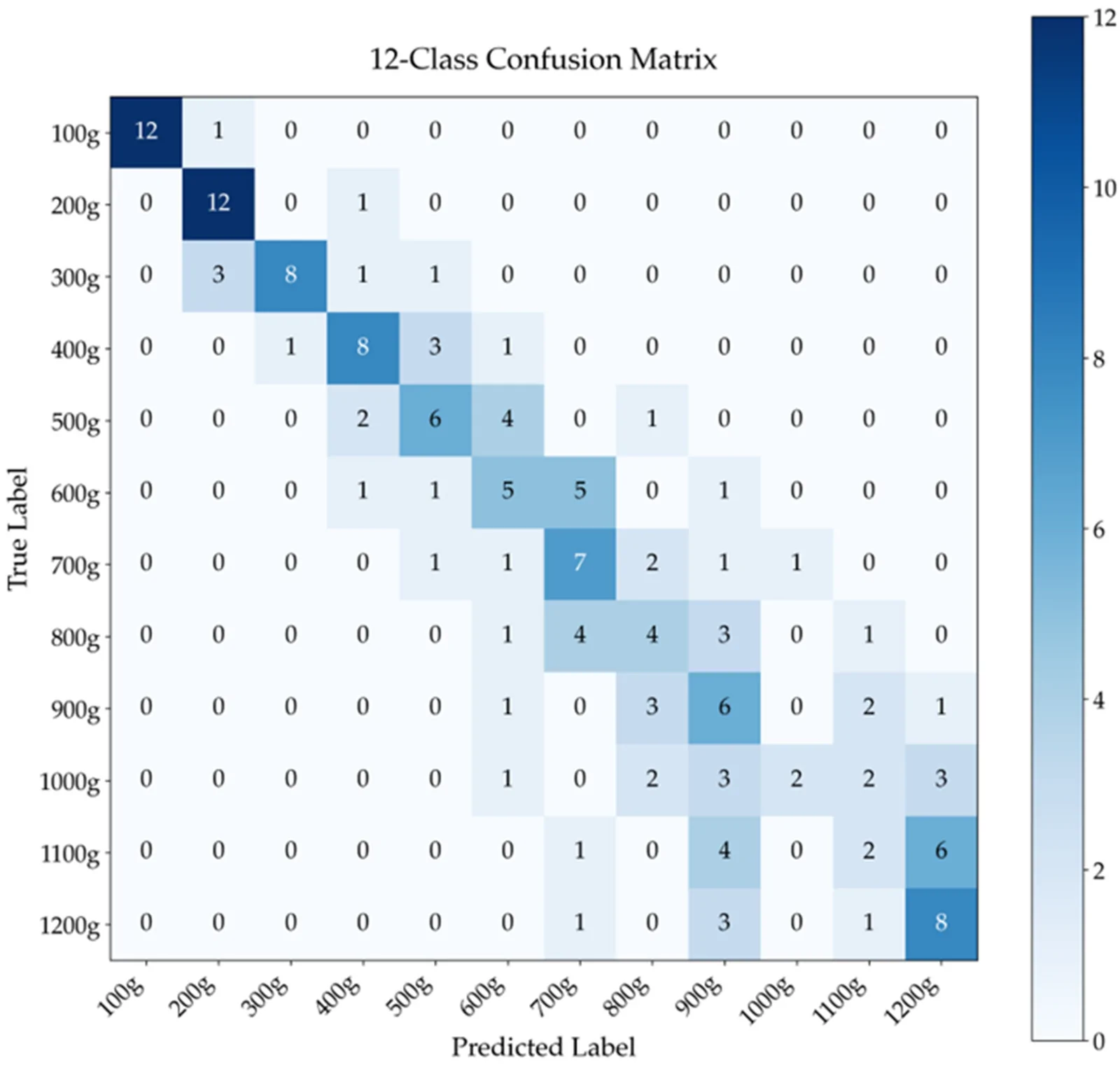
Confusion matrix for the 12-class CNN weight classifier.

Under the leakage-aware group-level split, the ResNet-18 CNN achieved an overall accuracy of 51.28% in the 12-class weight classification framework, which is 6.2 times greater than the random baseline (8.33%), with a macro-F1 score of 0.5051. Mapping predicted class labels back to gram values yields an MAE of 78.2 g and a within-±10% rate of 59%. Per-class recall declined with applied load: low weights (i.e., 100–500 g) were recovered most reliably (46–92%), mid-range weights (i.e., 600–800 g) at 31–54%, and high weights (i.e., 900–1100 g) were the hardest (15–46%). The highest class (1200 g) recovered to 61.5%, likely due to the distinctively saturated heatmap pattern at the extreme of the sensor’s dynamic range. The confusion matrix for the 12-class weight classifier is shown in Figure 6.

Table 3 presents the data on the performance of each classification method. The handcrafted-feature classical models performed substantially worse than the CNN on weight classification: the strongest, SVM (RBF), reached only 33.33% accuracy (macro-F1 = 0.3150, MAE = 123.7 g), followed by Random Forest (31.41%), XGBoost (26.92%), Extra Trees (25.64%), and kNN (21.79%). All classical models exhibited significantly greater errors than the CNN (MAE 124–183 g compared to 78 g). This gap—in contrast to the parity observed on the sector task—indicates that compact handcrafted descriptors capture the coarse spatial layout of a bite but not the fine-grained intensity differences required to discriminate applied load at 100 g resolution, which the end-to-end CNN learns directly from the heatmaps.

On the 12-class weight task, the end-to-end CNN reduced the MAE to 78 g—a reduction of roughly one third relative to the best classical model (124 g)—and raised its macro-F1 by about 60% (0.505 versus 0.315). The residual error of approximately 78 g, concentrated at the high loads where the heatmap response saturates, represents the practical limit of what the present heatmap representation can support for force quantification.

The main advantage of the end-to-end fine-tuned ResNet-18 over classical approaches for weight prediction is its ability to learn features. Compared with constant descriptors generated from the scaled bite-force map, the ResNet-18 network adapts its convolutional filters to the statistical features of the fiber-optic maps during training, retaining fine-grained information about intensity and gradients. Three CNN properties are relevant. First, its layered receptive fields include both the local peak intensity and the overall size of the pressure footprint—and both of these correlate with the applied load. Second, transfer learning from ImageNet provides a strong start for the early convolutional filters, allowing end-to-end training even with only about 713 training images. Third, the errors of the model are ordinally structured: the most frequent misclassifications are in the classes adjacent to the true weight, which results in a significantly lower MAE (78 g vs. 124–183 g) in comparison to classical models.

#### 3.2.2. Regression-Based Continuous Weight Prediction

**Figure 7:**
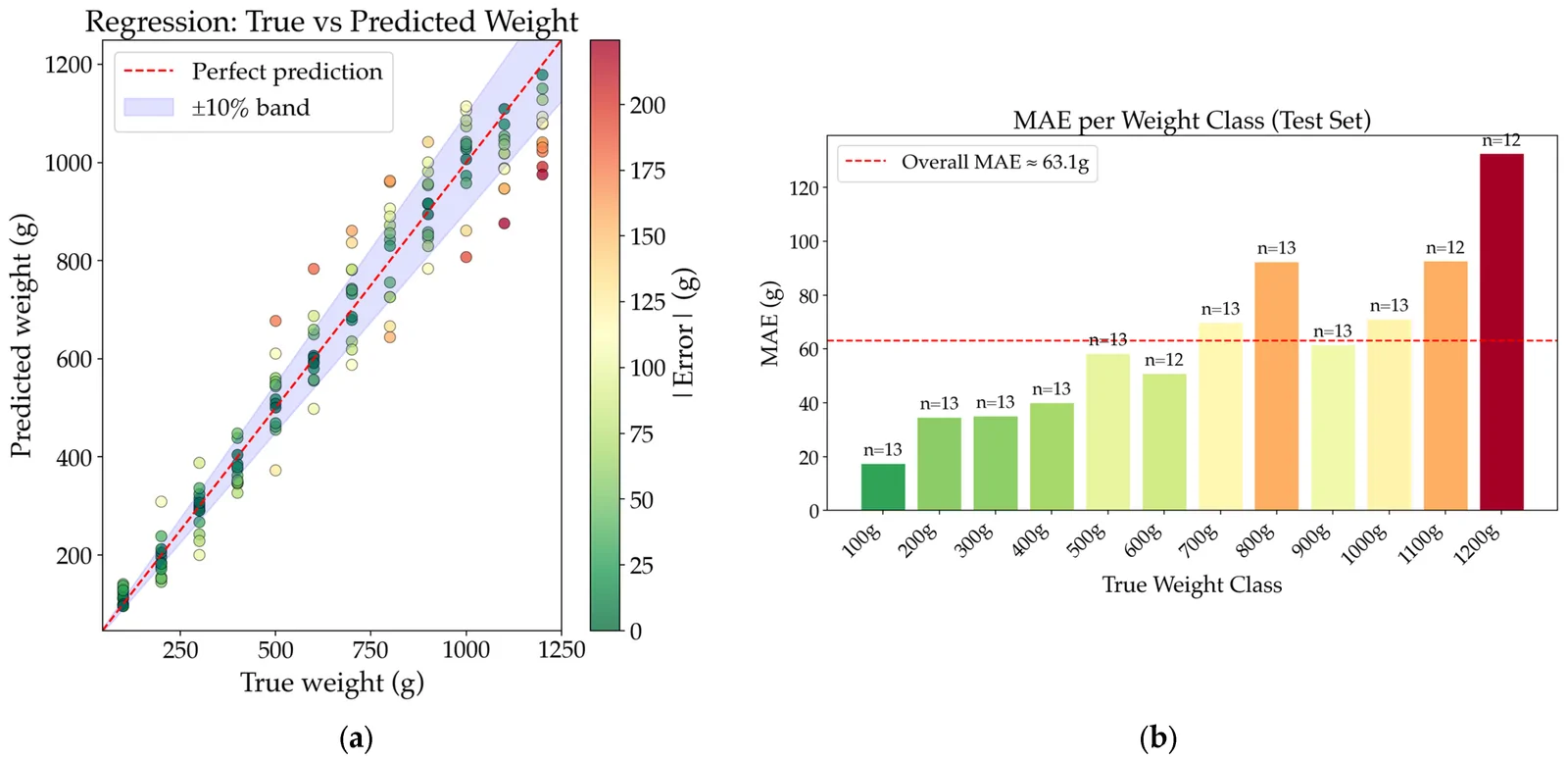
Regression-based continuous weight prediction on the held-out test set: (**a**) predicted versus true weight, with the identity line and the ±10% tolerance band; (**b**) MAE per weight class; the dashed line indicates the mean of the per-class MAE values.

The regression-based ResNet-18 version was evaluated on the same group-level test set. The model produced an MAE of 62.6 g, an RMSE of 81.6 g, and 54.9% of predictions were within ±10% of the true weight. Direct regression lowered the MAE by nearly 20% relative to the classification-derived error (MAE = 78.2 g), corresponding to a drop from around 6.5% to 5.2% of the 1200 g measurement range, and provided continuous outputs, not limited to the twelve discrete load levels. Figure 7a displays the predicted vs. real weight for the test set. The predictions cluster along the identity line across the entire 100–1200 g range, with most samples falling within the ±10% tolerance band. The MAE for each weight class is shown in Figure 7b, with the dashed line representing the mean of the per-class MAE values (≈63 g).

In the per-class classification, the absolute error grows with the applied load, from around 17 g for 100 g (34–40 g for the 200–400 g classes) up to 92–133 g for the largest loads (800–1200 g). This trend is in line with the saturation of the heatmap response for high loads, as observed in the classification experiments. The signed-error distribution is roughly symmetric for low and moderate loads. For the greatest loads, the distribution swings toward underestimation, in accordance with the compression of the sensor dynamic range. The rate of the regression model within ±10% (54.9%) is close to the value of the classification model (59%). This reflects that a relative ±10% criterion is a strict absolute bound at low loads, where it corresponds to only ±10 g at 100 g. In absolute terms, the regression errors at low loads are still small. The comparison between the two formulations is summarized in Table 4.

The key practical difference is in the resolution of the output, where the classification model is limited to twelve discrete levels of 100 g, while the regression model gives continuous force estimates across the whole range. The two formulations have similar error levels (~60–80 g), but the regression variant is more appropriate for continuous bite-force monitoring, because it delivers a finer, quantitative force reading.

Unlike the sector-localization task, where compact handcrafted features slightly exceeded the CNN, weight prediction clearly benefits from end-to-end feature learning, since it depends on fine-grained intensity differences rather than sector spatial layout.

## 4. Conclusions

This paper presents an ML-assisted distributed fiber optic sensor on an SLMux approach for high-resolution bite force analysis. The measurements were taken on 88 sensing points along the dental arch. The backscattered pattern was analyzed and transformed into a 2D map of wavelength-shift values. The results indicate that ML significantly enhances the interpretability of distributed sensing data. Under a leakage-aware group-level evaluation, on the 4-class sector-localization task, a handcrafted-feature classifier (Logistic Regression and SVM, both at 98.71%) performed slightly better than the end-to-end fine-tuned ResNet-18 CNN, which reached 98.06% accuracy. On the harder 12-class weight task, the CNN clearly outperformed every classical model, reaching 51.28% accuracy and a mean absolute error of 78 g (about 7% of the measurement range), reducing the error by roughly one third relative to the best classical model. Moreover, a regression-based ResNet-18 model trained to output continuous biting force values reduced test MAE to 62.6 g (RMSE = 81.6 g, ≈5% of the measurement range), a 20% error reduction over classification-based prediction. These results confirm the effectiveness of end-to-end fine-tuning in capturing the spatial and intensity patterns of the map based on the distributed fiber optic sensing system, while showing that coarse localization can also be solved with lightweight classical models trained on handcrafted features.

Nevertheless, the system has limitations in terms of low accuracy at a high level of pressure and with a small dataset. In addition, calibration was based on static point loads on a defined grid, which does not reproduce dynamic biting and real occlusal conditions. This study was built as a controlled proof-of-concept to test the sensing principle and machine learning pipeline under repeatable, well-characterized conditions. This required static point loading on a defined grid, providing exact weight and position values to calibrate the sensor per position and to train and evaluate the models. This controlled baseline is a critical first step before adding the unpredictability of dynamic and in vivo data.

Future work will involve improving the sensor design, increasing the dataset with more diverse and anatomically realistic occlusal samples, extending the experiments to multiaxial loading cases that reproduce different occlusal patterns of the dental jaw, and exploring new systems to improve the performance of dental bite-distributed sensing systems. Moreover, the research will focus on dynamic biting studies that mimic occlusal conditions, moving toward in vivo validation for clinical dentistry.

This study presents the feasibility of high-resolution bite force detection based on distributed fiber-optic sensing combined with ML techniques. The system is scalable and holds potential for being exploited in dental diagnostics, occlusal analysis, and individualized treatment approaches, and can be extended to rehabilitation activities.

## Figures and Tables

**Table 1:** Summary of ML experimental configurations.

Aspect	Sector Classification	Weight Prediction
Tasks	4-class	12-class classification, Regression
CNN Backbone	ResNet-18 (pretrained)	ResNet-18 (pretrained)
CNN Architecture	ResNet-18 (heatmap input)	Classification head
Classical ML models	LogReg, SVM, RF, XGBoost, kNN	SVM, RF, Extra Trees, XGBoost, kNN
Dataset	1019 images; group-level 70/15/15 split by grid cell	1019 images; group-level 70/15/15 split by grid cell
Primary Metrics	Accuracy, Macro-F1	Accuracy, Macro-F1, MAE (g)

**Table 2:** Consolidated comparison of all models on sector classification.

Model	4-Class Acc.	4-Class F1
Logistic Regression	98.71%	0.9869
SVM (RBF)	98.71%	0.9861
CNN (ResNet-18)	98.06%	0.9807
Random Forest	98.06%	0.9805
XGBoost	95.48%	0.9520
kNN	90.97%	0.8990

**Table 3:** Weight classification (12-class): CNN versus handcrafted-feature classical models on the group-level test set. CV Macro-F1 is the GroupKFold cross-validation score obtained during hyperparameter tuning (not applicable to the CNN).

Model	Accuracy	Macro-F1	MAE (g)	CV Macro-F1
ResNet-18 CNN	51.28%	0.5051	78.21	—
SVM (RBF)	33.33%	0.3150	123.72	0.3377
Random Forest	31.41%	0.2921	162.82	0.2413
XGBoost	26.92%	0.2515	164.10	0.2581
Extra Trees	25.64%	0.2504	175.00	0.2266
kNN	21.79%	0.2144	183.33	0.2074

**Table 4:** Weight prediction with ResNet-18 on the group-level test set: 12-class classification versus continuous regression.

Metric	12-Class CNN	Regression CNN
MAE (g)	78.21	62.57
RMSE (g)	—	81.62
Within ±10%	59%	54.9%
Output resolution	100 g (discrete)	Continuous

## Data Availability

The data are available upon reasonable request from the corresponding author.
